# An Exploratory Case Series Investigating Concurrent Aerobic and Resistance Training in Young, Highly Trained Rowers

**DOI:** 10.3390/sports14010039

**Published:** 2026-01-14

**Authors:** Melissa E. Brown, Angela L. Spence, Martyn J. Binnie, Dale W. Chapman

**Affiliations:** 1 Curtin School of Allied Health, Curtin University, Perth 6102, Australia; melissa.brown4@postgrad.curtin.edu.au (M.E.B.); angela.spence@curtin.edu.au (A.L.S.); 2 Curtin Medical Research Institute, Curtin University, Perth 6102, Australia; 3 Western Australian Institute of Sport, Perth 6010, Australia; mbinnie@wais.org.au

**Keywords:** concurrent training, ergometer performance, resistance training classification, rowing, training load, young athletes

## Abstract

This study examined the longitudinal patterns of concurrent aerobic and resistance training in young elite rowers to address the limited understanding of how training volume, modality, and periodisation interact across a season, and to introduce a novel rowing-specific resistance training classification. A retrospective design was used to analyse group training data over 36 weeks (*n* = 9; 20.6 ± 0.5 years), and individual case studies over 55 weeks (*n* = 4; 21.6 ± 0.4 years). Aerobic loads, resistance training tonnage, and ergometer performance (power output) were tracked, with resistance exercises categorised as rowing-specific, upper accessory, lower accessory, or core. Weekly aerobic volume averaged 14.0 ± 5.0 h, and rowing-specific resistance accounted for 48–57% of total tonnage (14.13 × 10^3^ ± 7.41 × 10^3^ kg). Exploratory analyses suggested an inverse relationship between aerobic, and resistance loads across training phases and trends toward improved ergometer power in three of four case athletes. High concurrent loads also appeared to coincide with occasional missed or modified sessions in several cases. These findings highlight the importance of managing concurrent loads to support consistent training while offering a practical resistance training classification that may enhance monitoring and decision-making for developing rowers.

## 1. Introduction

Rowing is a strength–power–endurance sport in which performance depends on the careful management of training frequency, volume, and intensity across both endurance and resistance training modalities [1,2,3]. Most aerobic work is completed below the lactate threshold, supplemented by targeted anaerobic sessions to meet racing demands [4,5,6]. Elite rowers may complete up to 14 concurrent sessions per week, accumulating approximately 18 h of aerobic training and up to 24 total weekly training hours [7,8]. Around two-thirds of this time is rowing-specific—on-water or ergometer-based—with the remainder involving resistance training and non-specific cross-training, such as cycling [8]. The integration of these modalities supports the force demands of rowing while contributing to musculoskeletal resilience and injury prevention [8,9].

Resistance training load is commonly quantified using total volume (tonnage), calculated as sets × repetitions × external load [9,10,11]. While tonnage provides a global indication of training stress, it does not capture exercise intent, specificity, or the differential contribution of individual exercises to rowing performance [10,11]. The contemporary literature highlights the need for load-monitoring frameworks that consider athlete characteristics and sport-specific demands [9]. To address this gap, the present study applies a novel resistance training classification that differentiates rowing-specific exercises from accessory upper-body, lower-body, and core movements, offering a more nuanced representation of resistance training stimuli [12].

Ergometer performance is widely used within national talent pathways to assess rowing capacity due to the environmental variability inherent in on-water testing [13,14]. Benchmark 2 km times and strength standards for key lifts such as the back squat, deadlift, and bench pull provide structured targets for athlete development [13]. Evidence suggests that resistance training performed at 72–92% of one-repetition maximum enhances hypertrophy, maximal strength, and power, with intensities rising to 85–95% of 1 RM during competition periods [4,8,15]. High-intensity interval training performed alongside resistance work can further improve maximal oxygen uptake and power output, reinforcing the importance of concurrent training for performance optimisation [16].

Despite these established principles, longitudinal data describing how young elite rowers balance concurrent loads across a full season remain limited. Furthermore, little research has examined how distinct resistance training exercise types contribute to load distribution or performance changes over time.

Therefore, this study aimed to (1) describe the volume, modalities, and periodisation of concurrent aerobic and resistance training across pre-season and competition phases in elite young rowers, and (2) explore associations between training patterns and ergometer performance through individual case analyses. Based on previous research, we hypothesised that periods characterised by higher concurrent aerobic and resistance training loads would be associated with improvements in submaximal and threshold ergometer power output, whereas sustained high loads may also coincide with reduced training consistency due to accumulated fatigue.

## 2. Materials and Methods

### 2.1. Study Design

This study employed a two-part retrospective longitudinal design consisting of (1) a group-level descriptive analysis and (2) four individual case studies. Data were collected across the 2022–2023 rowing season (June 2022–June 2023), with no deviation from athletes’ standard training. The season was divided into three phases:

- Pre-season (PRE; weeks 1–28): June to December, including routine monitoring and two 2 km ergometer tests (August and December).
- Domestic Competition (DOM; weeks 29–36): January to March, encompassing domestic racing and national selection trials, including the Australian National Championships (week 34).
- International Preparation and Competition (INT; weeks 37–55): March to June, including an additional 2 km ergometer selection test and final assessments prior to the U23 World Championships.

### 2.2. Participants

Nine athletes (four females, five males) contributed data during PRE and DOM, with four athletes continuing into INT. For de-identification purposes, athletes were assigned numerical identifiers (101–110) across the full cohort at the outset of data processing. All nine athletes contributed data during PRE and DOM; however, only four athletes (Athletes 101, 102, 104, and 105) were selected for international competition and therefore progressed into the INT phase and were included in the case series analyses. No athletes were excluded from the study or case series based on performance, injury, or outcome-related criteria. All athletes were scholarship or training-agreement holders at a state sport institute. Athlete calibre classifications followed the Participation Classification Framework [17]: Tier 4 athletes compete at national or international levels with more advanced performance standards, whereas Tier 3 athletes are progressing toward these benchmarks. Participant characteristics and performance profiles are summarised in Table 1.

Three athletes were identified as single-scull proficient (3.5 ± 1.2 training years), and six specialised in sweep rowing (2.6 ± 1.9 training years). Training age was defined as years of structured endurance training ≥ 8 h per week [18]. Written consent for retrospective data use was provided as part of each athlete’s scholarship agreement. The study received approval from the host institution’s Human Research Ethics Committee (HRE2023-0223) and adhered to the Declaration of Helsinki.

### 2.3. Training Data Collection

**Figure 1:**
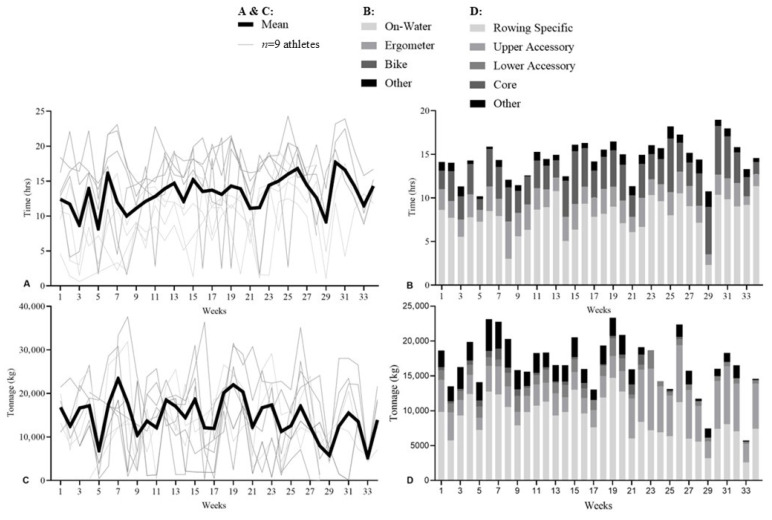
Aerobic and resistance training characteristics for all athletes (*n* = 9) across the PRE and DOM phases. (**A**): Mean (solid line) weekly aerobic training volume (hours) with individual patterns (grey line). (**B**): Distribution of aerobic training modalities (% time on on-water rowing, ergometer, cycling, and other). (**C**): Weekly resistance training tonnage (×10^3^ kg). (**D**): Mean (solid line) distribution of resistance training modalities (% tonnage for rowing-specific, upper accessory, lower accessory, core, and miscellaneous exercises) with individual distributions (grey line).

Resistance training sessions were prescribed and recorded using TeamBuildr (Silver Spring, MD, USA) and BridgeAthletic (San Francisco, CA, USA), with all data centrally stored in Smartabase (Fusion Sport, Brisbane, Australia). Aerobic training data—including heart rate (HR), distance, and duration—were collected via Garmin Forerunner 735XT devices (Garmin Ltd., Marsden Park, NSW, Australia) and synchronised through Garmin Connect to TrainingPeaks (TrainingPeaks LLC, Louisville, CO, USA). An overview of weekly aerobic and resistance training volumes and modalities for the cohort (*n* = 9) is presented in Figure 1.

Continuous HR monitoring during both ergometer and on-water sessions was obtained using Wahoo Tickr chest-strap monitors (Wahoo Fitness, Atlanta, GA, USA). Blood lactate concentrations were assessed using the Lactate Pro 2 analyser (Arkay Inc., Kyoto, Japan) immediately following T2 and THR ergometer sessions, as well as during monthly physiological assessments throughout PRE and DOM. Values were manually entered into a secure Excel database.

**Figure 2:**
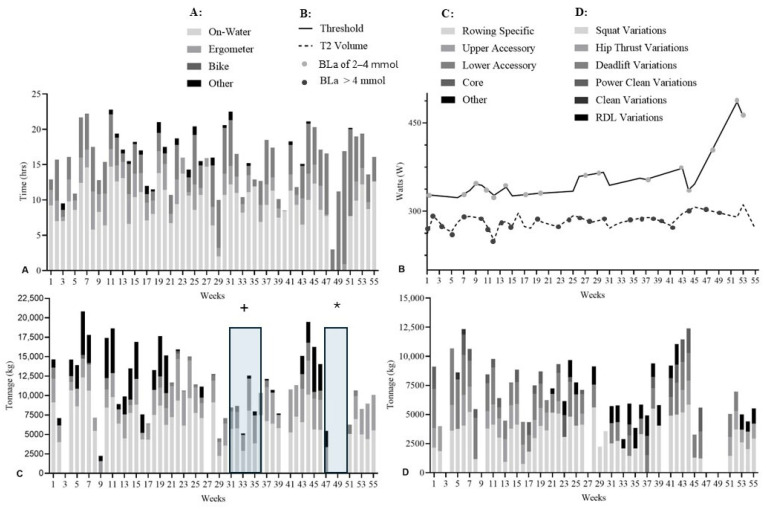
Training data for Athlete 101 across the PRE, DOM, and INT phases. (**A**): Aerobic training volume (hours) and modality distribution. (**B**): Ergometer power output (W) during T2 efforts (BLa-1 < 2.5 mmol) and THR efforts (BLa-1 > 4 mmol). (**C**): Resistance training tonnage (×10^3^ kg) and modality distribution; + indicates period of knee injury and * lumbar spine injury. (**D**): Rowing-specific sub-group exercises and corresponding tonnage (×10^3^ kg).

**Figure 3:**
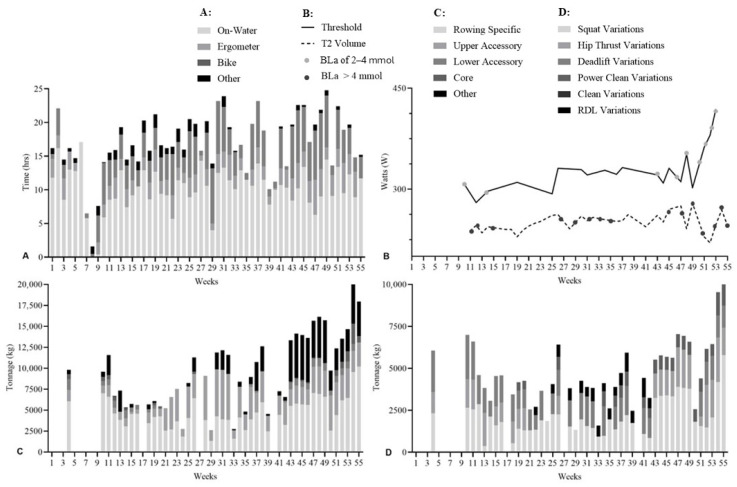
Training data for Athlete 102 across the PRE, DOM, and INT phases. (**A**): Aerobic training volume (hours) and modality distribution. (**B**): Ergometer power output (W) during T2 sessions (BLa-1 < 2.5 mmol) and THR sessions (BLa-1 > 4 mmol). (**C**): Resistance training tonnage (×10^3^ kg) and modality distribution. (**D**): Rowing-specific sub-group exercises and corresponding tonnage (×10^3^ kg).

**Figure 4:**
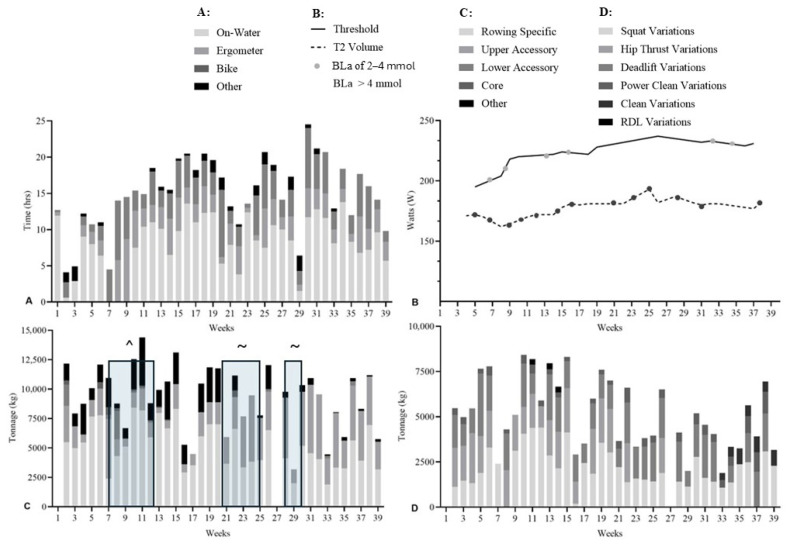
Training data for Athlete 104 across the PRE, DOM, and INT phases. (**A**): Aerobic training volume (hours) and modality distribution. (**B**): Ergometer power output (W) during T2 sessions (BLa-1 < 2.5 mmol) and THR sessions (BLa-1 > 4 mmol). (**C**): Resistance training tonnage (×10^3^ kg) and modality distribution; ^ indicates foot (impact) injury and ~ forearm/wrist injury. (**D**): Rowing-specific sub-group exercises and corresponding tonnage (×10^3^ kg).

**Figure 5:**
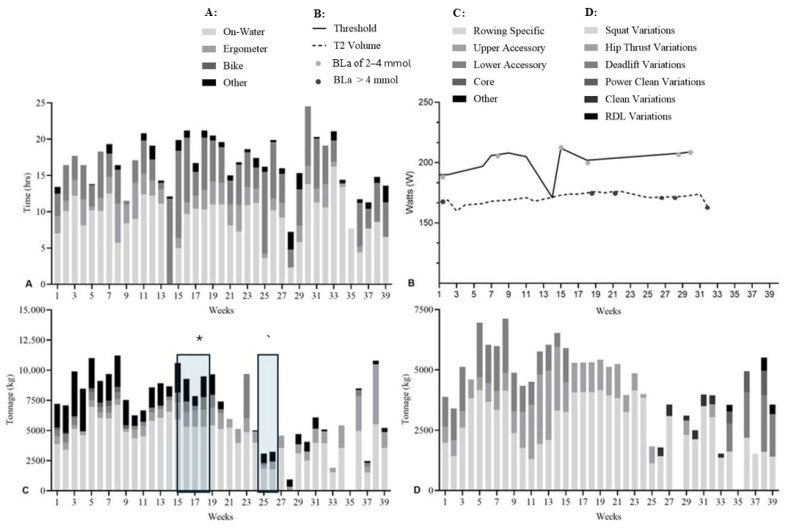
Training data for Athlete 105 across the PRE, DOM, and INT phases. (**A**): Aerobic training volume (hours) and modality distribution. (**B**): Ergometer power output (W) during T2 sessions (BLa-1 < 2.5 mmol) and THR sessions (BLa-1 > 4 mmol). (**C**): Resistance training tonnage (×10^3^ kg) and modality distribution; * indicates lumbar spine injury and ` chest wall injury. (**D**): Rowing-specific sub-group exercises and corresponding tonnage (×10^3^ kg).

Subjective internal load during ergometer training was assessed using the Borg RPE 6–20 scale [19]. Stroke rate (spm) and power output (W) were collected using Concept2 ergometers (Concept2, Morrisville, NC, USA) and exported to Excel for analysis. Weekly training adherence was monitored by coaching and performance staff, with sessions classified as missed (not completed) or modified (partially completed). Injury occurrences and affected anatomical regions were recorded descriptively to contextualise interruptions to training exposure, with injury timing indicated in graphical outputs (Figure 2, Figure 3, Figure 4 and Figure 5); however, injury incidence, severity, and causal relationships were not predefined outcomes of the study and were not analysed.

### 2.4. Resistance Training Classification and Processing

All aerobic and resistance training data were exported as separate CSV files and organised by training modality. Due to the high frequency of sessions, weekly aggregates were calculated for each athlete, including total tonnage (kg × 10^3^), training time (h), sets, and repetitions. Data aggregation was conducted in RStudio (2023.06.2+561 “Mountain Hydrangea” [20], descriptive statistics were generated in Jamovi 2.3.21 (Jamovi Project, 2023), and figures were produced using GraphPad Prism v10 (GraphPad Software, San Diego, CA, USA).

Across the season, 523 unique resistance training exercises were prescribed. Exercises were pre-categorised within TeamBuildr and BridgeAthletic and further refined in consultation with strength and conditioning practitioners. Classification was based on primary biomechanical role and intended training emphasis, resulting in five categories (Table 2):

- Rowing-specific compound lifts: deadlift variations, back and front squat, Romanian deadlift, power clean, clean pull, and other hip–trunk dominant movements central to rowing-specific force production.
- Upper accessory exercises: bench pull, bent-over row, lat pulldown, seated row, bench press, and dumbbell pressing movements.
- Lower accessory exercises: split squat, step-up, hip thrust, leg press, hamstring curl, and calf raise.
- Core exercises: plank variations, Pallof press, rotational medicine ball drills, and loaded carries.
- Miscellaneous/stability exercises: banded activation, bodyweight-assisted circuits, Swiss-ball tasks, and medicine ball conditioning.

Rowing-specific exercises were defined as compound lifts that most closely replicate the mechanical demands of the rowing stroke through hip–trunk force transfer. Accessory and core exercises were considered supportive for holistic development and injury mitigation rather than direct contributors to rowing-specific power. Exercises without quantifiable external load—such as banded and instability-based tasks—were grouped as “miscellaneous,” consistent with best-practice recommendations for resistance training load monitoring in applied settings [10].

Strength changes were extrapolated from maximal resistance sets using the Epley equation: 1 RM = Weight × (1 + 30/Reps), selected for its minimal tendency to overestimate strength and reduced residual error. This prediction model was selected due to its lower tendency to overestimate maximal strength and its reduced residual error compared with alternative formulae [21].

### 2.5. Ergometer Performance Measures

Ergometer power output was selected as the primary performance measure due to its established validity and reliability in assessing rowing capacity [22]. Longitudinal performance data were obtained from two weekly ergometer sessions representing distinct physiological targets.

Threshold 2 (T2) Aerobic Volume Sessions: T2 sessions consisted of 2 × 30 min continuous efforts at 18 spm. Athletes were encouraged to maximise sustainable power output while maintaining post-session blood lactate values below 2.5 mmol·L^−1^. This session type emphasised development of lactate threshold 1 (LT1) and aerobic endurance, consistent with contemporary endurance training models [18].

Threshold (THR) Anaerobic Power Sessions: THR sessions required rowing at higher stroke rates with the intent to exceed established power benchmarks. Athletes targeted post-session blood lactate concentrations above 4 mmol·L^−1^, corresponding to lactate threshold 2 (LT2). These sessions aimed to improve anaerobic capacity, fatigue tolerance, and high-intensity power output relevant to race demands [18].

Together, T2 and THR sessions provided complementary performance indicators, characterising aerobic efficiency, anaerobic work capacity, and phase-specific adaptation across the training season.

### 2.6. Statistical Analysis

All statistical analyses were conducted in RStudio (version 2020.06.1; RStudio Team, 2020) using established packages, including ggplot2 (version 3.4.0) for visualisation, tidyverse (version 1.3.1) as a meta-package suite, dplyr (version 1.1.0) for data manipulation, and readr (version 2.1.2) for data import. Aerobic and resistance training variables were summarised as mean ± standard deviation (SD), or as mean with range (minimum–maximum), consistent with the descriptive nature of the dataset.

Given the small sample size and the exploratory purpose of the study, a paired-samples t-test was used to examine within-athlete changes between PRE and DOM phases for aerobic training hours and resistance tonnage. In alignment with recommendations for exploratory analyses in high-performance sport contexts with restricted sample sizes, statistical significance was set at *p* < 0.10 to reduce the risk of Type II error. However, to avoid overinterpretation, all inferential outcomes are framed as trends rather than definitive effects, and interpretation emphasises effect sizes (ESs) and confidence intervals (CIs) rather than *p*-values alone.

Cohen’s *d* ESs were calculated and interpreted using conventional thresholds: trivial (<0.2), small (0.2–0.5), moderate (0.5–0.8), large (0.8–1.2), and very large (>1.2). Ninety-five percent confidence intervals (95% CIs) were included to contextualise the magnitude and precision of observed trends.

For the case series of selected athletes (*n* = 4), no inferential statistics were performed due to insufficient sample size. Instead, GraphPad Prism version 10 (GraphPad Software, San Diego, CA, USA, 2023) was used to produce customised visualisations depicting each athlete’s longitudinal training loads and performance across PRE, DOM, and INT preparation phases. The training phases were structured with a 2 km monitoring rowing ergometer performance at the end of each phase. While differences in the length of reported longitudinal training loads are due to athlete selection to World Championship crews (i.e., weeks 40–55). These visual analyses support individual-level interpretation of training–performance relationships where traditional group-level statistics are not appropriate.

## 3. Results

### 3.1. Group Results (n = 9)

Average aerobic training hours were higher during DOM compared with PRE (ES = 2.45; 95% CI: 2.24–2.65; *p* < 0.001). Weekly aerobic volume increased from 13.4 ± 5.2 h (range: 0.0–24.3) in PRE to 14.6 ± 5.2 h (range: 1.0–24.5) in DOM (Figure 1A). Aerobic training modalities were similar across phases, with PRE comprising 58% on-water rowing, 14% ergometer, 23% cycling, and 5% other; DOM consisted of 59% on-water rowing, 10% ergometer, 25% cycling, and 6% other (Figure 1B).

Total weekly resistance tonnage was higher in PRE than DOM (ES = 1.95; 95% CI: 1.75–2.15; *p* < 0.001). PRE tonnage peaked in week 16 (16.13 × 10^3^ ± 7.93 × 10^3^ kg) and was lowest in week 4 (8.22 × 10^3^ ± 8.68 × 10^3^ kg) (Figure 1C). DOM tonnage peaked in week 31 (12.13 × 10^3^ ± 6.90 × 10^3^ kg) and was lowest in week 28 (4.51 × 10^3^ ± 3.61 × 10^3^ kg).

Distribution of resistance training tonnage differed between phases. PRE consisted of 57% rowing-specific, 26% upper accessory, 6% lower accessory, 1% core, and 11% other exercises, whereas DOM consisted of 48% rowing-specific, 46% upper accessory, 2% lower accessory, and 4% other (Figure 1D).

Training adherence varied across PRE, with three athletes completing zero aerobic hours in weeks 4, 5, 6, and 15, and six athletes recording zero resistance tonnage across nine PRE weeks (1, 2, 3, 5, 6, 7, 8, 9, and 15). No athlete missed aerobic training in DOM, although four athletes recorded zero resistance tonnage in week 27.

### 3.2. Case Series (n = 4)

#### 3.2.1. Athlete 101

A male single-scull athlete (21.7 years; 192 cm; 100 kg; training age 3.9 years). Aerobic training averaged 16.2 ± 3.58 h/144 ± 33.0 km in PRE, 15.5 ± 4.42 h/140 ± 38.0 km in DOM, and 15.3 ± 4.80 h/108 ± 56.5 km in INT (Figure 2A).

T2 ergometer power increased across phases (PRE: 279 ± 11 W; DOM: 282 ± 7 W; INT: 293 ± 14 W), with an average stroke rate of 18 ± 1 spm (Figure 2B). THR power also increased (PRE: 336 ± 11 W at 24 ± 1 spm; DOM: 355 ± 9 W at 24 ± 1 spm; INT: 400 ± 69 W at 27 ± 5 spm).

Rowing-specific resistance training comprised 59.8% (PRE), 59.3% (DOM), and 56.0% (INT) of total tonnage. Experienced injuries in INT (weeks 31–37 and 47–51) (Figure 2C). In this athlete the 2 km ergometer performance showed a 1.6% and 0.8% improvement in PRE, no change in DOM, and a 0.6% improvement in INT (Figure 2D).

#### 3.2.2. Athlete 102

A male single-scull athlete (21.7 years; 180 cm; 84 kg; training age 4.5 years). Aerobic training averaged 15.9 ± 4.47 h/138 ± 45.5 km in PRE, 18.7 ± 4.23 h/150 ± 37.8 km in DOM, and 18.1 ± 4.22 h/152 ± 31.6 km in INT (Figure 3A).

T2 power increased from PRE to DOM (244 ± 9 W to 255 ± 3 W) and remained similar in INT (253 ± 18 W) (Figure 3B). THR power increased across phases (PRE: 303 ± 17 W; DOM: 326 ± 5 W; INT: 343 ± 39 W).

Rowing-specific resistance training accounted for 60.6% (PRE), 44.6% (DOM), and 44.7% (INT) of tonnage (Figure 3C). This athlete achieved their highest relative strength in DOM and highest resistance tonnage in INT. For this athlete their 2 km ergometer performance improved by 1.8% (PRE) and 2.5% (DOM) but declined by 14.2% in INT (Figure 3D).

#### 3.2.3. Athlete 104

A female sweep specialist (21 years; 180 cm; 72.4 kg; training age 2.2 years). Aerobic training averaged 14.6 ± 4.67 h/118 ± 44.3 km in PRE, 16.6 ± 5.52 h/129 ± 44.4 km in DOM, and 11.9 ± 3.04 h/113 ± 31.8 km in INT (Figure 4A).

T2 power increased from PRE to DOM (244 ± 9 W to 255 ± 3 W) and remained stable in INT (253 ± 18 W) (Figure 4B). THR power increased across phases (PRE: 303 ± 17 W; DOM: 326 ± 5 W; INT: 343 ± 39 W).

Rowing-specific resistance training accounted for 58.4% (PRE), 48.5% (DOM), and 54.8% (INT) of total tonnage (Figure 4C). This athlete experienced injuries in PRE (weeks 7–12) and DOM (weeks 20–26 and 28–30), which corresponded with a plateau in strength and reduced resistance tonnage. 2 km ergometer results showed a 1.4% decrease and 4.2% increase in PRE, a 2.5% improvement in DOM, and no INT test (Figure 4D).

#### 3.2.4. Athlete 105

A female single-scull athlete (22 years; 173 cm; 72.3 kg; training age 3.4 years). Aerobic training averaged 16.9 ± 3.25 h/123 ± 39.5 km in PRE, 16.2 ± 5.46 h/125 ± 41.5 km in DOM, and 14.2 ± 0.85 h/70.5 ± 17.7 km in INT (Figure 5A).

T2 power remained stable from PRE to DOM (170 ± 5 W to 169 ± 6 W) and was not measured in INT (Figure 5B). THR power increased from PRE to DOM (199 ± 12 W to 209 ± 1 W), with no INT data.

Rowing-specific resistance training accounted for 61.6% (PRE), 67.8% (DOM), and 60.2% (INT) of tonnage (Figure 5C). This athlete sustained injuries in PRE (weeks 14–18) and DOM (weeks 24–26), reducing total resistance tonnage but increasing rowing-specific work and relative strength during DOM and INT. The 2 km ergometer performance changed by −2.2% and +3.7% in PRE, improved by 3.7% in DOM, and was not tested in INT (Figure 5D).

## 4. Discussion

This study provides a season-long examination of concurrent aerobic and resistance training in young elite rowers and contributes to the limited longitudinal literature describing real-world training practices within high-performance development environments. The aerobic training volumes observed across the season were broadly consistent with previous reports in elite rowing cohorts, where weekly endurance training commonly exceeds 17 h and is dominated by rowing-specific modalities [8,23]. Such high-volume, low-intensity training remains foundational for the optimisation of oxidative capacity, lactate kinetics, and technical economy. The alignment of the present findings with national performance benchmarks further underscores the entrenched reliance on high aerobic loads within talent pathways and highlights the necessity of systematic monitoring to optimise both performance and athlete health [7,13].

A major contribution of this study is the introduction of a structured classification system for resistance training exercises, distinguishing rowing-specific movements from upper accessory, lower accessory, and core exercises. This framework represents an advancement in rowing-specific load quantification because traditional tonnage-based reporting fails to integrate the biomechanical specificity and functional role of individual exercises. The categorisation of rowing-specific movements—primarily squats, deadlifts, and Olympic lifting derivatives—reflects their direct contribution to the hip–trunk force production central to rowing performance. Accessory exercises, by contrast, provide complementary strength, stability, and injury-prevention benefits but do not directly replicate rowing force vectors or neuromuscular recruitment patterns.

This refined categorisation mirrors contemporary trends in team sports and endurance disciplines, where load quantification has shifted from volume-centric to functionally informed models capable of capturing the complexity of modern training practice [24,25]. Furthermore, the potential integration of this framework with systems-modelling approaches—such as Banister-type impulse-response models or emerging machine-learning methods—may significantly enhance coaches’ ability to predict performance trajectories, manage fatigue, and individualise training prescription [26]. While the present study adopts this classification descriptively, future applications may include evaluating dose–response relationships or predicting maladaptation and injury based on deviations in rowing-specific resistance loading.

Across the training year, the observed fluctuations in weekly aerobic and resistance loads revealed an undulating pattern with limited evidence of a rigidly applied periodisation structure. Early PRE resembled a block periodisation model characterised by concentrated strength development, before transitioning into a more undulating and competition-oriented approach. This practical, emergent pattern may reflect the constraints of applied high-performance environments, where training is influenced by environmental conditions, athlete availability, illness, injury, and the competing priorities of technical and physical development. The notable reduction in resistance tonnage during DOM—coinciding with intensified technical preparation and competition demands—aligns with previous evidence suggesting that aerobic readiness and technical quality may take precedence over strength development during phases of competitive racing [27,28]. These findings highlight the dynamic nature of concurrent training in elite rowing and call into question the extent to which traditional linear or block periodisation models are feasible or appropriate within youth high-performance settings.

The case series component offers further insight into the interaction between individual athlete characteristics and training adaptation. Although aerobic training loads were relatively homogenous, resistance training loads—particularly rowing-specific components—varied substantially between athletes. Such variation likely reflects deliberate individualisation based on technical needs, positional demands, and injury histories [10,15,29]. Athletes with higher training age demonstrated greater stability in load tolerance and training continuity, consistent with previous research indicating that accumulated training experience enhances both physiological robustness (e.g., improved collagen turnover, neuromuscular resilience, and recovery kinetics) and psychological preparedness [12,27,30].

Associations between rowing-specific resistance training and ergometer performance improvements, observed in several athletes, align with evidence supporting high-intensity strength training (72–92% 1 RM progressing to 85–95% 1 RM) to target force–velocity characteristics relevant to rowing performance [4,15,31]. These findings also support literature advocating personalised resistance training prescription—rather than uniform squad-based programming—to optimise technical efficiency, power output, and long-term athlete development [13,32,33]. Although the present study was not powered to detect statistical relationships between training patterns and performance outcomes, descriptive trends suggest that fluctuations in rowing-specific resistance training may exert a meaningful influence on competitive readiness, particularly when integrated with high-quality aerobic and threshold-based training. Future research may extend the present framework by integrating on-water performance metrics derived from global positioning systems (GPS), inertial measurement units (IMUs), or emerging combined sensor platforms. Such technologies enable the assessment of stroke kinematics, boat velocity, and performance variability under ecologically valid conditions, as demonstrated in rowing and paddle sports contexts [34,35]. Integrating these measures with longitudinal training load data may provide deeper insight into how concurrent aerobic and resistance training prescriptions translate to technical efficiency and race-specific performance outcomes.

Collectively, the present findings underscore the complexity of managing concurrent training in rowing. Balancing the competing demands of aerobic development, strength enhancement, technical training, recovery optimisation, and injury mitigation remains challenging, particularly within youth elite cohorts. The classification system proposed in this study provides a foundational framework that may support more precise load distribution, enhance interdisciplinary communication, and reduce ambiguity regarding the contribution of resistance training to rowing performance. Future studies integrating internal load measures (e.g., HRV, RPE, hormonal markers), biomechanical outputs, and performance modelling would help elucidate the multi-layered interactions underpinning adaptation and performance across the rowing season.

### 4.1. Limitations

This study’s limitations stem primarily from its small sample size and retrospective observational design, which limit statistical inference and generalisability. The reliance on training management systems also introduces variability due to missing or inconsistently recorded data, particularly for exercises not suited to traditional tonnage quantification. Additionally, planned training data were unavailable for comparison with actual training behaviours; therefore, analyses reflect emergent training patterns rather than coaching intent. The classification of resistance exercises, while grounded in practical coaching frameworks, necessarily involved subjective judgement and may vary across coaches, programs, or sporting contexts, which could limit reproducibility of the classification approach in different settings. Despite these constraints, the study contributes rare season-long insight into training loads within a high-performance rowing development program and lays groundwork for more sophisticated monitoring frameworks.

### 4.2. Practical Applications

The resistance training classification proposed in this study provides a practical tool for coaches seeking to quantify and monitor strength-training contributions within concurrent rowing programmes. By distinguishing rowing-specific from accessory loading, coaches can refine exercise selection, improve periodisation sequencing, and better align strength development with technical and physiological goals. This approach enhances the functional interpretation of training load, which is often obscured by traditional tonnage metrics, and may inform more nuanced athlete monitoring, communication, and readiness strategies. Further validation across different athlete populations and integration with internal load metrics will strengthen its utility within high-performance systems.

## 5. Conclusions

This study offers a comprehensive account of training load distribution, exercise composition, and periodisation across a competitive season in young elite rowers. The novel resistance training classification provides a more contextually relevant approach to quantifying strength-training contributions within a concurrent training model, advancing traditional load-monitoring practices. The findings highlight the importance of individualised programming, nuanced load management, and precise monitoring to optimise adaptation, manage injury risk, and support long-term athlete development. Future research incorporating systems modelling, internal load metrics, and planned-vs-actual training comparisons will further elucidate the mechanisms underpinning training adaptation in elite rowing pathways.

## Figures and Tables

**Table 1:** Athlete characteristics and performance capacity descriptive measures mean ± standard deviation (SD) for all athletes (*n* = 9) and case series (*n* = 4).

Analysis	Sex	*n*	Age (Years)	Weight (kg)	Height (m)	2 km Ergometer (min: s)	$\dot{V}$O_2_max (L/min)
Group							
	Male	5	20.4 ± 1.9	92.7 ± 7.9	1.88 ± 0.04	6:06 ± 0.1	5.50 ± 0.3
	Female	4	20.9 ± 0.8	71.9 ± 2.6	1.77 ± 0.06	6:59 ± 0.5	3.80 ± 0.2
Case series							
	Male	2	21.7 ± 0.0	92.0 ± 11.3	1.98 ± 0.06	5:59 ± 2.1	5.70 ± 0.3
	Female	2	21.5 ± 0.7	72.9 ± 0.6	1.76 ± 0.04	7:07 ± 0	3.96 ± 0.1

**Table 2:** Resistance training exercise groups classification.

Category	Strength	Power	Stability
Rowing Specific			
	Clean Pull Deadlifts GHD Extension Hip Thrust RDLs Squats (Back, Front, Overhead, SL)	Power Clean	
Upper Body			
	Bench Press Bench Pull Bicep Curl Overhead Press Rows Triceps Extension		
Lower Body			
	Calf Raises Hip Abduction Hip Thrust Leg Curl Leg Extension Lunges Nordic Curl Step Ups	Lunges, step-ups (explosive with speed)	
Core			
			Dead Bug Leg Raises Pallof Press Planks Russian Twists

GHD = glute-hamstring developer, SL = single leg, RDL = Romanian deadlift.

## Data Availability

The data used in this study contain confidential athlete information and are not publicly available. De-identified datasets may be provided by the corresponding author upon reasonable request and with permission from the sport institute.
